# Supplementary material for: Variation of Female Pronucleus Reveals Oocyte or Embryo Chromosomal Copy Number Variations
Source: Adv Genet (Hoboken). 2022 Nov 10;4(1):2200001. doi: 10.1002/ggn2.202200001 (PMC10000260; doi:10.1002/ggn2.202200001)
Supplement: Supplementary file 1 — Supporting Information [file GGN2-4-2200001-s001.pdf]

ADVANCED GENETICS
Open Access

Supporting Information

for *Advanced Genetics*, DOI 10.1002/ggn2.202200001

Variation of Female Pronucleus Reveals Oocyte or Embryo Chromosomal Copy Number Variations

Jingwei Yang, Yikang Wang, Chong Li, Wei Han, Weiwei Liu, Shun Xiong, Qi Zhang, Keya Tong, Guoning Huang and Xiaodong Zhang**

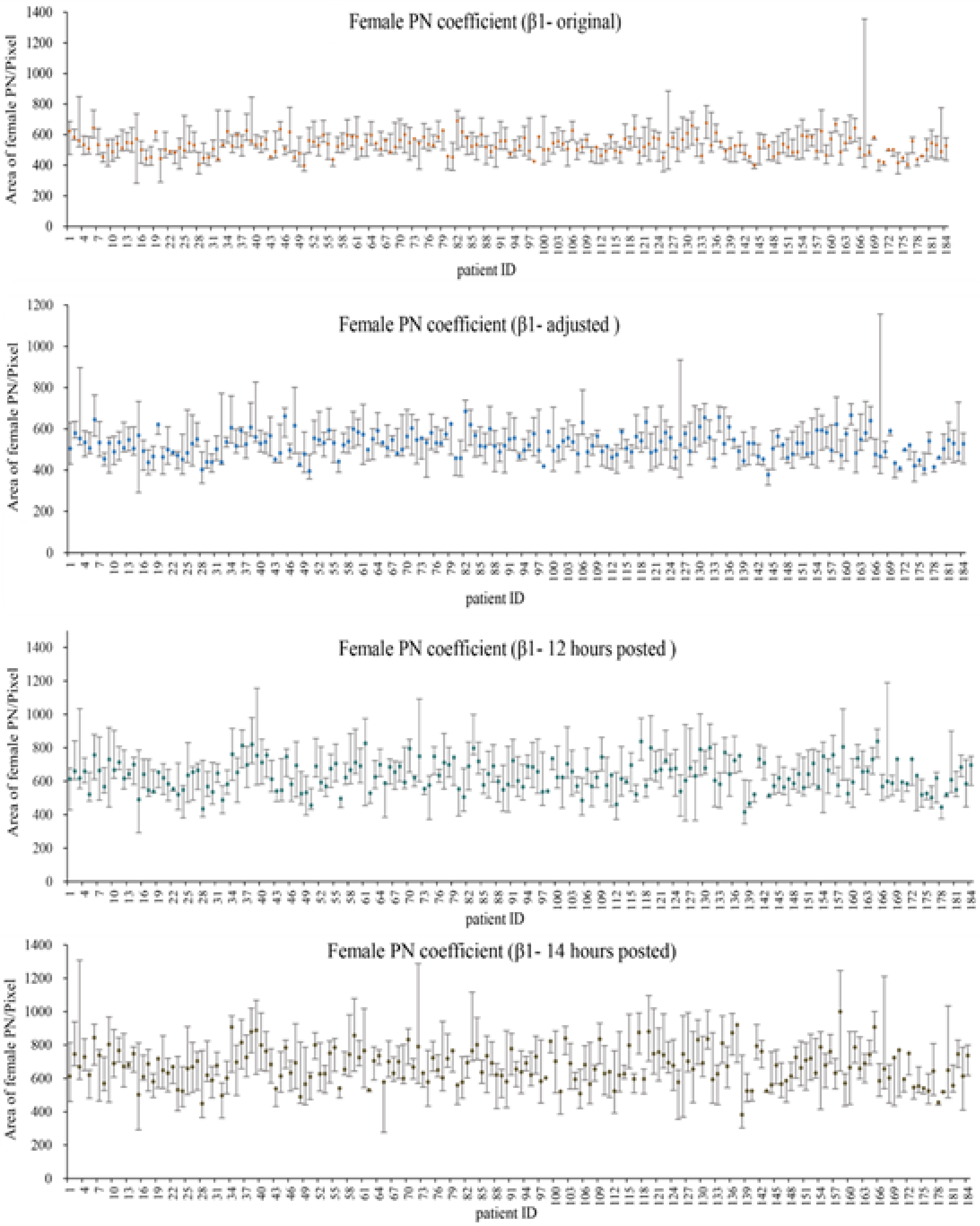

Fig. S1 Heterogeneity of female pronucleus coefficient β1.

Number of embryo	Ranking										
2	1	2									First 1
3	1	2	3								First 2
4	1	2	3	4							Medi
5	1	2	3	4	5						Last 2
6	1	2	3	4	5	6					Last 1
7	1	2	3	4	5	6	7				
8	1	2	3	4	5	6	7	8			
9	1	2	3	4	5	6	7	8	9		
10	1	2	3	4	5	6	7	8	9	10	
11	1	2	3	4	5	6	7	8	9	10	11

Fig. S2 Definition of ranking by PN coefficient β1.

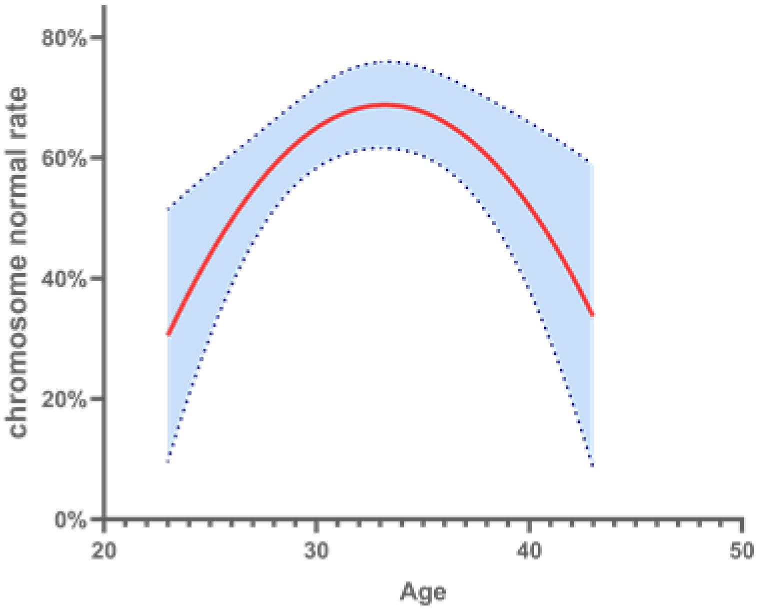

Fig. S3 Chromosome-normal rate in the female age-dependent distribution.

Table S1-1. Finding a chromosome-normal blastocyst by the original ranking of embryos.

Number of embryos	*n*	First	Median 1	Last	First 1 & 2	Median 2	Last 1 & 2
2	34	11/17 (64.71)	/	7/17 (41.18)	/	/	/
3	93	16/31 (51.61)	14/31 (45.16)	18/31 (58.06)	/	/	/
4	104	11/26 (42.31)	26/52 (50.00)	10/26 (38.46)	21/52 (40.38)	/	26/52 (50.00)
5	125	12/25 (48.00)	36/75 (48.00)	10/25 (40.00)	25/50 (50.00)	13/25 (52.00)	20/50 (40.00)
6	114	15/19 (78.95)	42/76 (55.26)	7/19 (36.84)	28/38 (73.68)	19/38 (50.00)	17/38 (44.74)
7	84	11/12 (91.67)	39/60 (65.00)	8/12 (66.67)	21/24 (87.50)	20/36 (55.56)	17/24 (70.83)
8	96	8/12 (66.67)	40/72 (55.56)	6/12 (50.00)	14/24 (58.33)	27/48 (56.25)	13/24 (54.17)
9	9	0/1 (0)	3/7 (42.86)	0/1 (0)	1/2 (50.00)	2/5 (40.00)	0/2 (0)
10	60	3/6 (50.00)	32/48 (66.67)	3/6 (50.00)	6/12 (50.00)	25/36 (69.44)	7/12 (58.33)
11	22	1/2 (50.00)	8/18 (33.33)	1/2 (50.00)	2/4 (50.00)	6/14 (42.86)	2/4 (50.00)
12	36	2/3 (66.67)	10/30 (33.33)	0/3 (0)	3/6 (50.00)	8/24 (33.33)	1/6 (16.67)
13	13	0/1 (0.00)	7/11 (63.64)	0/1 (0)	0/2 (0.00)	7/9 (77.78)	0/2 (0)
Total	790	90/155 (58.06)*	227/480 (47.29)	70/155 (45.16)*	121/204 (59.31)#	127/235 (54.04)	103/204 (50.49)#

*58.06% vs. 45.16%, OR = 1.68 (1.07–2.64), *P* = 0.031

#59.31% vs. 50.49%, OR = 1.43 (0.97–2.14), *P* = 0.091

Table S1-2. Finding a chromosome-normal blastocyst by the adjusted ranking of embryos.

Number of embryos	*n*	First	Median 1	Last	First 2	Median 2	Last 2
2	34	10/17 (58.82)		8/17 (47.06)	/	/	/
3	93	17/31 (54.84)	13/31 (41.94)	18/31 (58.06)	/	/	/
4	104	12/26 (46.15)	27/52 (51.92)	8/26 (30.77)	21/52 (40.38)	/	26/52 (50.00)
5	125	13/25 (52.00)	35/75 (46.67)	10/25 (40.00)	25/50 (50.00)	14/25 (56.00)	19/50 (38.00)
6	114	14/19 (73.68)	42/76 (55.26)	8/19 (42.11)	26/38 (68.42)	21/38 (55.26)	17/38 (44.74)

7	84	11/12 (91.67)	39/60 (65.00)	8/12 (66.67)	20/24 (83.33)	21/36 (58.33)	17/24 (70.83)
8	96	8/12 (66.67)	40/72 (55.56)	6/12 (50.00)	15/24 (62.50)	26/48 (54.17)	13/24 (54.17)
9	9	0/1 (0)	3/7 (42.86)	0/1 (0)	0/2 (0)	3/5 (60.00)	0/2 (0)
10	60	3/6 (50.00)	32/48 (66.67)	3/6 (50.00)	6/12 (50.00)	25/36 (69.44)	7/12 (58.33)
11	22	1/2 (50.00)	8/18 (44.44)	1/2 (50.00)	1/4 (25.00)	7/14 (50.00)	2/4 (50.00)
12	36	2/3 (66.67)	10/30 (33.33)	0/3 (0)	3/6 (50.00)	8/24 (33.33)	1/6 (16.67)
13	13	0/1 (0)	7/11 (63.64)	0/1 (0)	0/2 (0)	7/9 (77.78)	0/2 (0)
Total	790	91/155 (58.71)*	246/480 (51.25)	70/155 (45.16)*	117/204 (57.35)#	132/235 (56.17)	102/204 (50.00)#

*58.71% vs. 45.16%, OR = 1.73 (1.10–2.71), $P = 0.023$

#57.35% vs. 50.00%, OR = 1.35 (0.91–1.99), $P = 0.164$

Table S1-3. Finding a chromosome-normal blastocyst by the ranking of 12-h embryos.

Number of embryos	*n*	First	Median 1	Last	First 2	Median 2	Last 2
2	34	11/17 (64.71)		7/17 (41.18)	/	/	/
3	93	19/31 (61.29)	14/31 (45.16)	15/31 (48.39)	/	/	/
4	104	13/26 (50.00)	26/52 (50.00)	8/26 (30.77)	26/52 (50.00)	/	21/52 (40.38)
5	125	15/25 (60.00)	34/75 (45.33)	9/25 (36.00)	27/50 (54.00)	12/25 (48.00)	19/50 (38.00)
6	114	14/19 (73.68)	42/76 (55.26)	8/19 (42.11)	25/38 (65.79)	20/38 (52.63)	19/38 (50.00)
7	84	12/12 (100.00)	39/60 (65.00)	7/12 (58.33)	22/24 (91.67)	20/36 (55.56)	16/24 (66.67)
8	96	9/12 (75.00)	35/72 (48.61)	10/12 (83.33)	14/24 (58.33)	27/48 (56.25)	13/24 (54.17)
9	9	1/1 (100.00)	2/7 (28.57)	0/1 (0)	1/2 (50.00)	2/5 (40.00)	0/2 (0)
10	60	4/6 (66.67)	32/48 (66.67)	2/6 (33.33)	10/12 (83.33)	22/36 (61.11)	6/12 (50.00)
11	22	0/2 (0)	9/18 (50.00)	1/2 (50.00)	2/4 (50.00)	5/14 (35.71)	3/4 (75.00)
12	36	2/3 (66.67)	10/30 (33.33)	0/3 (0)	3/6 (50.00)	9/24 (37.50)	0/6 (0)
13	13	0/1 (0)	7/11 (63.64)	0/1 (0)	0/2 (0)	7/9 (77.78)	0/2 (0)

Total	790	100/155 (64.52)*	250/480 (52.08)	67/155 (43.23)*	130/204 (63.73)#	124/235 (52.77)	97/204 (47.55)#

*64.52% vs. 43.23%, OR = 2.39 (1.51–3.77), $P < 0.001$

#63.73% vs. 47.55%, OR = 1.94 (1.30–2.88), $P = 0.001$

Table S1-4. Finding a chromosome-normal blastocyst by the ranking of 14-h embryos.

Number of embryos	*n*	First	Median 1	Last	First 2	Median 2	Last 2
2	34	11/17 (64.71)		7/17 (41.18)	/	/	/
3	93	21/31 (67.74)	11/31 (35.48)	16/31 (51.61)	/	/	/
4	104	13/26 (50.00)	25/52 (48.08)	9/26 (34.62)	26/52 (50.00)	/	21/52 (40.38)
5	125	13/25 (52.00)	36/75 (48.00)	9/25 (36.00)	26/50 (52.00)	11/25 (44.00)	21/50 (42.00)
6	114	17/19 (89.47)	39/76 (51.32)	8/19 (42.11)	28/38 (73.68)	17/38 (44.74)	19/38 (50.00)
7	84	11/12 (91.67)	40/60 (66.67)	7/12 (58.33)	21/24 (87.50)	23/36 (63.89)	14/24 (58.33)
8	96	9/12 (75.00)	38/72 (52.78)	7/12 (58.33)	14/24 (58.33)	26/48 (54.17)	14/24 (58.33)
9	9	1/1 (100.00)	2/7 (28.57)	0/1 (0)	1/2 (50.00)	2/5 (40.00)	0/2 (0)
10	60	5/6 (83.33)	31/48 (64.58)	2/6 (33.33)	9/12 (75.00)	24/36 (66.67)	5/12 (41.67)
11	22	0/2 (0)	9/18 (50.00)	1/2 (50.00)	2/4 (50.00)	6/14 (42.86)	2/4 (50.00)
12	36	2/3 (66.67)	10/30 (33.33)	0/3 (0)	3/6 (50.00)	8/24 (33.33)	1/6 (16.67)
13	13	0/1 (0)	7/11 (63.64)	0/1 (0)	1/2 (50.00)	5/9 (55.56)	1/2 (50.00)
Total	790	103/155 (66.45)*	248/480 (51.67)	66/155 (42.58)*	131/204 (64.22)#	122/235 (51.91)	98/204 (48.04)#

*66.45% vs. 42.58%, OR = 2.61 (1.68–4.24), $P < 0.001$

#64.22% vs. 48.04%, OR = 1.94 (1.31–2.89), $P = 0.001$.

Table S2. PGT results and female PN 14 h coefficient β1 ranking.

PGT result	First 1	First 2	Median	Last 2	Last 1
Chromosome-normal and mosaic	103/155 (66.45%)*	60/107 (56.07%)	133/266 (50%)	55/107 (51.4%)	66/155 (42.58%)*
Euploid with errors	22/155 (14.19%)#	26/107 (24.3%)	63/266 (23.68%)	30/107 (28.04%)	45/155 (29.03%)#
Aneuploid with errors	30/155 (19.35%)	21/107 (19.63%)	70/266 (26.32%)	22/107 (20.56%)	44/155 (28.39%)

*66.45% vs. 42.58%, OR = 2.67 (1.68–4.24), $P < 0.001$

#14.19% vs. 29.03%, OR = 0.40 (0.23–0.71), $P = 0.002$

Table S3. PGT results (error stratification) and female PN 14 h coefficient β1 ranking.

PGT result	First 1	First 2	Median	Last 2	Last 1
Chromosome-normal	72/155 (46.45%)*	43/107 (40.19%)	100/266 (37.59%)	43/107 (40.19%)	44/155 (28.39%)*
Sole mosaic	31/155 (20.00%)	17/107 (15.89%)	33/266 (12.41%)	12/107 (11.21%)	22/155 (14.19%)
Sole deletion and/or duplication	15/155 (9.68%)#	17/107 (15.89%)	41/266 (15.41%)	19/107 (17.76%)	31/155 (20.00%)#
Sole deletion and/or duplication euploidy embryos with mosaic forms	9/155 (5.81%)	12/107 (11.21%)	22/266 (8.27%)	11/107 (10.28%)	14/155 (9.03%)
Sole aneuploidy	16/155 (10.32%)	11/107 (10.28%)	37/266 (13.91%)	11/107 (10.28%)	25/155 (16.13%)
Aneuploidy embryos with mosaic forms	7/155 (4.52%)	2/107 (1.87%)	22/266 (8.27%)	8/107 (7.48%)	7/155 (4.52%)
Aneuploidy embryos with deletion and/or duplication	2/155 (1.29%)	2/107 (1.87%)	6/266 (2.26%)	2/107 (1.87%)	5/155 (3.23%)
Aneuploidy embryos with chromosomal deletion and/or duplication and mosaic forms	3/155 (1.94%)	3/107 (2.8%)	5/266 (1.88%)	1/107 (0.93%)	7/155 (4.52%)

*46.45% vs. 28.39%, OR = 2.19 (1.37 – 3.50), $P < 0.001$

#9.68% vs. 20%, OR = 0.43 (0.22–0.83), $P = 0.016$

Table S4. PGT-A results and female PN 14 h coefficient β1 ranking.

PGT-A result	First 1	First 2	Median	Last 2	Last 1
Chromosome-normal and mosaic	54/81 (66.67%)	36/56 (64.29%)	73/136 (53.68%)	34/57 (59.65%)	42/81 (51.85%)
Euploid with errors	12/81 (14.81%)	8/56 (14.29%)	28/136 (20.59%)	9/57 (15.79%)	18/81 (22.22%)
Aneuploid with errors	15/81 (18.52%)	12/56 (21.43%)	35/136 (25.74%)	14/57 (24.56%)	21/81 (25.93%)

Table S5. PGT-A results (error stratification) and female PN 14 h coefficient β1 ranking.

PGT-A result	First 1	First 2	Median	Last 2	Last 1
Chromosome-normal	42/81 (51.85%)	25/57 (43.86%)	51/136 (37.5%)	30/57 (52.63%)	28/81 (34.57%)
Sole mosaic	12/81 (14.81%)	11/57 (19.3%)	22/136 (16.18%)	4/57 (7.02%)	14/81 (17.28%)
Sole deletion and/or duplication	7/81 (8.64%)	6/57 (10.53%)	16/136 (11.76%)	5/57 (8.77%)	13/81 (16.05%)
Sole deletion and/or duplication euploidy embryos with mosaic forms	6/81 (7.41%)	3/57 (5.26%)	12/136 (8.82%)	4/57 (7.02%)	5/81 (6.17%)
Sole aneuploidy	9/81 (11.11%)	6/57 (10.53%)	28/136 (20.59%)	7/57 (12.28%)	11/81 (13.58%)
Aneuploidy embryos with mosaic forms	3/81 (3.7%)	2/57 (3.51%)	5/136 (3.68%)	5/57 (8.77%)	4/81 (4.94%)
Aneuploidy embryos with deletion and/or duplication	1/81 (1.23%)	2/57 (3.51%)	2/136 (1.47%)	1/57 (1.75%)	6/81 (7.41%)
Aneuploidy embryos with chromosomal deletion and/or duplication and mosaic forms	1/81 (1.23%)	2/57 (3.51%)	0	0	0

Table S6. PGT-SR results and female PN 14 h coefficient β1 ranking.

PGT-SR result	First 1	First 2	Median	Last 2	Last 1
Chromosome-normal and mosaic	49/74 (66.22%)*	24/50 (48%)	60/130 (46.15%)	21/50 (42%)	24/74 (32.43%)*
Euploid with errors	10/74 (13.51%)#	18/50 (36%)	35/130 (26.92%)	21/50 (42%)	27/74 (36.49%)#
Aneuploid with errors	15/74 (20.27%)	8/50 (16%)	35/130 (26.92%)	8/50 (16%)	23/74 (31.08%)

*66.22% vs. 32.43%, OR = 4.08 (2.06–8.10), $P < 0.001$

#13.51% vs. 36.49%, OR = 0.27 (0.12–0.61), $P = 0.001$

Table S7. PGT-SR results (error stratification) and female PN 14 h coefficient β1 ranking.

PGT-SR result	First 1	First 2	Median	Last 2	Last 1
Chromosome-normal	30/74 (40.54%)*	18/50 (36.00%)	49/130 (37.69%)	13/50 (26.00%)	16/74 (21.62%)*
Sole mosaic	19/74 (25.68%)#	6/50 (12.00%)	11/130 (8.46%)	8/50 (16.00%)	8/74 (10.81%)#
Sole deletion and/or duplication	8/74 (10.81%)	11/50 (22.00%)	25/130 (19.23%)	14/50 (28.00%)	18/74 (24.32%)

Sole deletion and/or duplication euploidy embryos with mosaic forms	3/74 (4.05%)	9/50 (18.00%)	10/130 (7.69%)	7/50 (14.00%)	9/74 (12.16%)
Sole aneuploidy	7/74 (9.46%)	5/50 (10.00%)	20/130 (15.38%)	4/50 (8.00%)	14/74 (18.92%)
Aneuploidy embryos with mosaic forms	4/74 (5.41%)	0	6/130 (4.62%)	3/50 (6.00%)	3/74 (4.05%)
Aneuploidy embryos with deletion and/or duplication	1/74 (1.35%)	0	4/130 (3.08%)	0	4/74 (5.41%)
Aneuploidy embryos with chromosomal deletion and/or duplication and mosaic forms	2/74 (2.7%)	1/50 (2%)	5/130 (3.85%)	1/50 (2%)	2/74 (2.7%)

*40.54% vs. 21.62%, OR = 2.47 (1.20–5.09), *P* = 0.020

#25.68% vs. 10.81%, OR = 2.85 (1.16–7.01), *P* = 0.032

Table S8. PGT-SR (errors from the proband) results by female PN 14 h coefficient β1 ranking.

PGT-SR result	First 1	First 2	Median	Last 2	Last 1
Chromosomal normal embryo	26/42 (61.9%)*	10/27 (37.04%)	32/73 (43.84%)	12/27 (44.44%)	10/42 (23.81%)*
Embryo's chromosomal error coincident with female	12/42 (28.57%)	13/27 (48.15%)	28/73 (38.36%)	10/27 (37.04%)	21/42 (50%)
Embryo's chromosomal error not coincident with female	4/42 (9.52%)	4/27 (14.81%)	13/73 (17.81%)	5/27 (18.52%)	11/42 (26.19%)

*61.9% vs. 23.81%, OR = 5.20 (2.02–13.37), *P* < 0.001

Table S9. PGT-SR results (errors from the proband) and male PN 14 h coefficient β1 ranking.

PGT-SR result	First 1	First 2	Median	Last 2	Last 1
Chromosomal normal embryo	19/32 (59.38%)	13/23 (56.52%)	28/57 (49.12%)	12/23 (52.17%)	16/32 (50%)
Embryo's chromosomal error coincident with male	9/32 (59.38%)	7/23 (30.43%)	23/57 (40.35%)	6/23 (26.09%)	6/32 (18.75%)
Embryo's chromosomal error not coincident with male	4/32 (12.5%)	3/23 (13.04%)	6/57 (10.53%)	5/23 (21.74%)	10/32 (31.25%)

Table S10. Female PN and total DNA content.

Rank	No change	Increase	Decrease	
1	89/155 (57.42%)	32/155 (20.64%)	33/155 (21.29%)	155
2	79/155 (50.97%)	40/155 (25.81%)	36/155 (23.23%)	155
3	78/138 (56.52%)	39/138 (28.26%)	21/138 (15.22%)	138

4	46/107 (42.99%)	25/107 (23.36%)	36/107 (33.64%)	107
5	40/81 (49.38%)	21/81 (25.93%)	20/81 (24.69%)	81
6	33/56 (58.93%)	12/56 (21.43%)	11/56 (19.64%)	56
7	23/37 (62.16%)	9/37 (24.32%)	5/37 (13.51%)	37
8	13/25 (52.00%)	8/25 (32.00%)	4/25 (16.00%)	25
9	8/13 (61.54%)	4/13 (30.77%)	1/13 (7.69%)	13
10	6/12 (50.00%)	3/12 (25.00%)	3/12 (25.00%)	12
>10	2/11 (18.18%)	5/11 (45.45%)	4/11 (36.36%)	11
	417	199	174	790

Correlation analysis: $P = 0.491$
